# Factors that impact a patient’s experience when undergoing single-photon emission computed tomography myocardial perfusion imaging (SPECT-MPI) in the US: A survey of patients, imaging center staff, and physicians

**DOI:** 10.1007/s12350-019-01863-0

**Published:** 2019-08-29

**Authors:** Junlong Li, David R. Walker, Ginger Biesbrock, Rita M. Kristy, Hongbo Yang, Emily Gao, Sarah Koenigsberg, James R. Spalding, Therese M. Kitt

**Affiliations:** 1 Analysis Group, Inc., 111 Huntington Avenue, 14th Floor, Boston, MA 02199 USA; 2 Astellas Pharma Global Development, Northbrook, IL USA; 3 MedAxiom, Neptune Beach, FL USA

**Keywords:** CAD, SPECT, MPI

## Abstract

**Background:**

Single-photon emission computed tomography myocardial perfusion imaging (SPECT-MPI) is commonly used for coronary artery disease diagnosis/assessment in the United States (US); however, the factors that most significantly affect patients’ experience when undergoing SPECT-MPI are not well known.

**Methods:**

In this US-based cross-sectional study, an online questionnaire was used to identify and quantify attributes of the SPECT-MPI process that impact patients’ experience, according to adults who underwent SPECT-MPI in the prior month, cardiac imaging center staff, and referring physicians. Participants were asked to rate the importance of 32 factors using an 11-point scale; congruence between groups (physicians vs patients, patients vs imaging center staff, and physicians vs imaging center staff) was assessed.

**Results:**

The survey was completed by 101 patients, 101 center staff, and 100 physicians, who gave similar ratings for the highest-rated factors (high-quality results/decreasing likelihood of having to retest, highly skilled and knowledgeable staff, and compassionate and respectful staff). Congruence was higher between patients and imaging center staff compared with physicians and patients, and was notably low between imaging center staff and physicians.

**Conclusions:**

We identified areas for improvement in the patient SPECT-MPI experience that could translate into improved quality and value.

**Electronic supplementary material:**

The online version of this article (10.1007/s12350-019-01863-0) contains supplementary material, which is available to authorized users.

## Introduction

Use of non-invasive single-photon emission computed tomography myocardial perfusion imaging (SPECT-MPI) in the diagnosis and assessment of coronary artery disease (CAD) among adults in the United States (US) is a well-established practice, with an estimated 7 to 8 million SPECT-MPI procedures performed annually.^1^-^3^ While ensuring a positive patient experience has been a priority among nuclear imaging centers that perform SPECT-MPI, there is a paucity of evidence-based recommendations for guiding improvements in the experience of patients undergoing nuclear imaging, particularly for SPECT-MPI. Of the few studies evaluating the patient experience with nuclear imaging, several have discussed methods by which imaging centers or radiology departments could make improvements to their facilities and/or services, such as offering longer hours of operation and free parking;^4^-^7^ however, insights from various perspectives into improving the patient experience are limited. One qualitative study found that patients are often inadequately informed before undergoing SPECT-MPI and present to the imaging center for testing feeling anxious as a result.^8^

The patient experience can be defined as a patient’s evaluation of their experience with the healthcare system, including both clinical and emotional interactions, which are collectively shaped by people, processes, and physical settings.^9^ A multitude of factors across the continuum of care can contribute to the patient experience and may ultimately affect patient outcomes. Therefore, data involving multiple stakeholder perspectives can help provide important insights that could guide efforts to deliver a better patient experience and improve care involving SPECT-MPI testing. Given their vital role in the SPECT-MPI process and their level of patient contact throughout the procedure, gaining insights from the imaging center staff is potentially critical for improving the patient experience. It is just as critical that referring physicians understand their patients’ potential concerns and the factors that patients deem important. Here, we report the results of a study designed to identify and evaluate factors of the SPECT-MPI testing process that impact the patient experience in the US. The specific primary objectives were to (1) identify major factors affecting the patient experience with SPECT-MPI and investigate the relative importance of these factors from the patients’ perspective; (2) investigate the relative importance of these factors to patient experience, as judged by physicians and imaging center staff; and (3) evaluate the congruence between the ratings of patients, physicians, and imaging center staff in terms of how each group rated the relative importance of these factors to the patient experience. As a secondary objective, in order to support the optimal delivery of care, we assessed the attributes of referring physicians/physicians’ offices that are preferred by nuclear imaging staff when patients are referred for SPECT-MPI.

## Methods

### Study Design and Eligibility

This US-based cross-sectional study included a qualitative phase and a quantitative phase. The qualitative phase consisted of a targeted literature review to identify factors that may potentially affect the patient experience of SPECT-MPI, followed by one-on-one phone interviews with patients, as well as imaging center staff and physicians, to refine the original list. An online survey was designed based on the results of the qualitative phase. In the quantitative phase, the importance of each identified factor was rated according to the perspectives of patients, imaging center staff, and referring physicians via the online survey.

Adult patients (aged 18 years or older) who were located in the US were eligible if they underwent a SPECT-MPI test on an outpatient basis at a nuclear imaging center or lab in the month prior to the interview or survey. Imaging center staff were required to serve in a leadership role or help operate a nuclear imaging center or lab that provides outpatient SPECT-MPI testing. Cardiovascular service line leaders, imaging or nuclear medical directors, and operations leaders for practice or cardiovascular diagnostic testing were eligible. Physicians were eligible to participate if they were a US-licensed cardiologist or primary care physician (PCPs) who had been practicing for at least 3 years and referred at least 5 patients for SPECT-MPI imaging on an outpatient basis in the 3 months prior to the interview or survey. All participants were required to speak English.

Recruiting was conducted via existing US panels in partnership with professional survey vendors: Schlesinger Associates for physicians and patients, and MedAxiom for center staff. Both organizations recruited respondents who met the specified eligibility criteria and consented to join the study. Demographic information for participants was used to assess representative sampling (e.g., on the basis of geographic region, race, gender).

The study was approved by the New England Institutional Review Board (NEIRB). Each participant was required to complete a NEIRB-approved informed consent document and was compensated at pre-established rates approved by the NEIRB for their participation in the study.

### Identification of Factors Influencing the Patient Experience

A targeted literature review was conducted to identify the factors that affect patients’ experience with SPECT-MPI. The targeted literature review, which included peer-reviewed publications and gray literature, was conducted using PubMed and web-based searches. The search utilized terms related to the patient experience, patient satisfaction, quality of care, nuclear imaging centers, SPECT-MPI, and radiology. An initial list of 27 factors were identified in literature as important to the patient experience when undergoing SPECT-MPI and categorized into 1 of 5 categories: communication and education, logistics and convenience, imaging center technologies, imaging center accessibility, and imaging center staff and atmosphere.

The initial list of 27 factors was then refined based on feedback obtained via 1-hour, one-on-one telephone interviews with 6 patients, 6 imaging center staff members, and 6 referring physicians (3 cardiologists and 3 PCPs). Interview participants gave feedback on factors of importance to patients’ experience with SPECT-MPI (based on, but not limited to, those in the initial list identified from the targeted literature review). As a result of the interviews, the original list of 27 factors expanded to a total of 32 factors.

### Assessing Importance and Congruency of Factors

#### Design of survey questionnaire

An online survey questionnaire was developed based on the finalized list of 32 factors from the phone interviews. Screening questions were included at the beginning of the questionnaire in order to verify the participant’s eligibility and collect data on participant characteristics (including demographics and experience with SPECT-MPI testing), and all surveys concluded with quality control questions. The majority of questions asked participants to rate the importance of each of the 32 patient experience factors using an 11-point scale (0-10; where 0 signified “not at all important” and 10 signified “extremely important”). Using an 11-point rating scale in the online survey promoted validity and reliability of measurement. Many studies in literature support the notion that validity and reliability is higher for scales with a moderate number of points, with some studies indicating reliability continued to increase up to lengths of 11 points.^14^ Therefore, by providing questions with 11-point scales, we were able to capture opinions with granularity without sacrificing reliability. Nine questions related to the care process were also included to further explore patients’ experience with nuclear imaging. For example, patients who received pharmacologic-induced (instead of exercise-induced) stress while undergoing SPECT-MPI were asked to select which pharmacologic agent they received; physicians were asked which pharmacologic stress agent they preferred, and center staff were asked which stress agent was most commonly used at their center. Responses available for each question included dobutamine (Dobutrex®), adenosine (Adenoscan®), regadenoson (Lexiscan®), dipyridamole (Persantine®), other/please specify, and do not know/unsure.

The order of rating questions within each category and the order of the five categories (those determined during the targeted literature review) were randomized over respondents to avoid potential effects due to respondent fatigue or other changes in attention throughout the course of the survey.

#### Survey launch and data collection

First, a series of pilot tests with 2 patients, 2 imaging center staff members, and 2 physicians was conducted to ensure the logic and clarity of the questionnaire. The questionnaire was revised based on feedback received during the pilot testing. After the pilot testing, a soft launch of the survey was conducted in order to recruit about 10% of the targeted 100 participants from each group. Soft launch data met quality check standards, with no adjustments made to the questionnaire before proceeding to the full launch.

After confirming the clarity and logic of the questionnaire, the survey was fully launched to recruit the targeted 100 participants from each group of interest. In the soft and full launches, eligible patients and physicians were randomly sampled from a panel representative of US geographic regions maintained by a survey vendor; eligible imaging center staff were recruited by a cardiovascular performance consulting group.

After the full launch was complete, data quality checks were conducted whereby respondents were flagged for providing identical ratings for all rating questions or failing to provide expected responses for quality control questions. Data from these respondents were not included in the data analysis. The data collected were de-identified to ensure that the opinions and information collected from participants could not be linked to personal identifying information.

### Data Analysis

Descriptive statistics of participant characteristics, the description of care processes, and rating scores for each factor were summarized for the three respondent groups: patients, imaging center staff, and physicians. Continuous variables were summarized using means, standard deviations, medians, and ranges, while categorical variables were described using counts and proportions.

To assess congruency between the median ratings of the groups, differences between rating scores for physicians vs patients, patients vs imaging center staff, and physicians vs imaging center staff were assessed using Wilcoxon rank-sum tests with Bonferroni correction.^10^ A *P* value <  0.017 (0.05/3 by applying the Bonferroni correction) was considered a statistically significant difference in importance rating between 2 participant groups. All statistical analyses were conducted using SAS version 9.4 (SAS Institute, Cary, NC).

## Results

### Participant Characteristics

Demographic characteristics for the 101 patients, 101 imaging center staff, and 100 physicians (50 cardiologists and 50 PCPs) who were randomly selected and completed the online survey are summarized in Table 1. Most patients and physicians were male (54.5% and 86.0%, respectively), whereas nearly two-thirds of center staff were female (63.4%), and most of the participants (> 70% per category) were white and non-Hispanic. While 54.5% of patients underwent SPECT-MPI testing in an urban facility and 54.5% of center staff worked at an urban facility, only 37.0% of physicians practiced in an urban area, and 53.0% practiced in a suburban area.

**Table 1:** Summary of demographic characteristics

Characteristics	Patients (N = 101)	Imaging center staff (N = 101)	Physicians (N = 100)
Sex, n (%)			
Male	55 (54.5%)	37 (36.6%)	86 (86.0%)
Female	46 (45.5%)	64 (63.4%)	14 (14.0%)
Age (years), mean (SD) [range]	54.7 (15.2) [19–87]	48.7 (9.7) [30–71]	54.1 (9.4) [34–72]
Race, n (%)			
White	78 (77.2%)	91 (90.1%)	71 (71.0%)
American Indian or Alaska Native	0 (0.0%)	1 (1.0%)	0 (0.0%)
Asian	2 (2.0%)	4 (4.0%)	21 (21.0%)
Native Hawaiian/Other Pacific Islander	0 (0.0%)	0 (0.0%)	1 (1.0%)
Black or African American	14 (13.9%)	3 (3.0%)	1 (1.0%)
Other or ≥ 2 races	6 (5.9%)	1 (1.0%)	3 (3.0%)
Unknown	1 (1.0%)	1 (1.0%)	3 (3.0%)
Ethnicity, n (%)			
Hispanic	12 (11.9%)	4 (4.0%)	4 (4.0%)
Non-Hispanic	88 (87.1%)	95 (94.1%)	94 (94.0%)
Unknown	1 (1.0%)	2 (2.0%)	2 (2.0%)
Geographic region, n (%)			
Northeast	31 (30.7%)	10 (9.9%)	38 (38.0%)
Midwest	13 (12.9%)	32 (31.7%)	22 (22.0%)
West	25 (24.8%)	16 (15.8%)	22 (22.0%)
South	32 (31.7%)	43 (42.6%)	18 (18.0%)
Geographic area,^a^ n (%)			
Urban	55 (54.5%)	55 (54.5%)	37 (37.0%)
Rural	12 (11.9%)	7 (6.9%)	10 (10.0%)
Suburban	34 (33.7%)	39 (38.6%)	53 (53.0%)

Key: SD, standard deviation

^a^Represents the geographic area of testing for patients, imaging center staff, and of practice for physicians

In addition to the characteristics shown in Table 1, nearly half (49.5%) of patients had received at least a bachelor’s degree, and 89.1% had at least some college education. Imaging center participants were directors/managers of nuclear imaging, directors/managers of non-invasive imaging, clinical operations directors, and practice managers. Additionally, imaging center participants were, in general, experienced operators, with an average of 10.9 years (range 1-35 years) in their role at their imaging centers, for which the average number of patients undergoing SPECT-MPI testing was 229 patients/month (range 40-1200). For physicians, the average number of years in practice was 21.1 years (range 5-37 years). There were 72 physicians who worked in private practice settings, 21 who practiced in community hospitals, and 7 who practiced in academic medical centers. On average, participating physicians had ordered a SPECT-MPI test for 54.3 unique patients (range 5-300) on an outpatient basis in the 90 days prior to the survey.

### Description of Care Process

The description of care process is summarized in the supplementary appendix (Tables S1, S2, S3). In brief, the information most commonly provided to patients prior to the test was information regarding what to expect during the test, how to prepare, and information regarding test location and directions to the nuclear imaging center. According to physicians and imaging center staff, many of their patients (68.0% and 43.6%, respectively) did not have access to test results independently before going over the results with their physician. A similar trend was observed among patients, with half of patients (50.5%) reporting that they were not granted independent access to results.

Regadenoson was the most preferred stress agent among physicians (56%) and the most commonly used by imaging center staff (94.1%). Imaging center preference (38.8%) and known side effects of the stress agent (33.7%) were most commonly cited by physicians as factors influencing the choice of stress agent; the choice stress agent among imaging center staff was predominantly influenced by side effects (58.5%) and cost (14.9%). Although most imaging center staff (74.3%) believed that patients were aware of which stress agent was used, the majority of patients (56.5%) reported being unaware of which agent had been used.

### Ratings of Factors That Influence the Patient Experience

**Figure 1:**
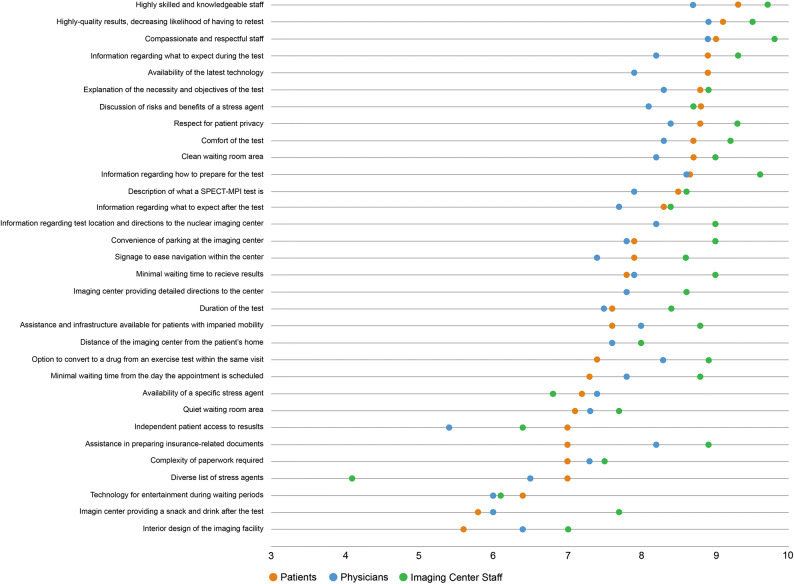
Average rating scores of importance for aspects of the SPECT-MPI process to the patient experience

The importance to the patient experience of 32 factors related to the SPECT-MPI process, as rated by patients, imaging center staff, and physicians in the online survey, is shown in Table 2 and Fig. 1. Patients ranked highly skilled and knowledgeable staff as the most important factor (median 10; mean 9.3), followed by high-quality results/decreasing likelihood of having to retest (median 10; mean 9.1), and compassionate and respectful staff (median 10; mean 9.0). Compassionate and respectful staff (median 10; mean 9.8) was rated as the most important factor by imaging center staff, followed by highly skilled and knowledgeable staff (median 10; mean 9.7), and information regarding how to prepare for the test (median 10; mean 9.6). Physicians considered high-quality results/decreasing likelihood of having to retest, and compassionate and respectful staff as the most important factors (median for both, 9; mean for both, 8.9) to their patients. Rating of the least important factor varied, with the lowest scores obtained for interior design of the facility (median 6; mean 5.6) for patients; diverse list of stress agents (median 4; mean 4.1) for imaging center staff; and independent patient access to results (median 6; mean 5.4) for physicians.

**Table 2:** Summary of importance (in 0-10 scale) for aspects of the SPECT-MPI process to the patient experience

Factor^a^	Patients			Imaging center staff			Physicians		
	N	Mean (SD)	Median (range)	N	Mean (SD)	Median (range)	N	Mean (SD)	Median (range)
Communication and education									
Information regarding what to expect during the test	101	8.9 (1.6)	10 [2–10]	101	9.3 (1.0)	10 [6–10]	100	8.2 (1.5)	8 [4–10]
Explanation of the necessity and objectives of the test	101	8.8 (1.7)	9 [2–10]	101	8.9 (1.4)	9 [4–10]	100	8.3 (1.6)	9 [3–10]
Information regarding how to prepare for the test	101	8.6 (2.2)	10 [0–10]	101	9.6 (1.0)	10 [5–10]	100	8.6 (1.4)	9 [3–10]
Description of what a SPECT-MPI test is*	101	8.5 (2.0)	9 [0–10]	101	8.6 (1.6)	9 [1–10]	100	7.9 (1.6)	8 [3–10]
Information regarding what to expect after the test	101	8.3 (2.5)	9 [0–10]	101	8.4 (2.1)	9 [1–10]	100	7.7 (1.8)	8 [2–10]
Information regarding test location and directions to the nuclear imaging center*	101	8.2 (2.4)	9 [0–10]	101	9.0 (1.7)	10 [1–10]	100	8.2 (1.6)	8 [4–10]
Independent patient access to results	101	7.0 (2.9)	8 [0–10]	101	6.4 (2.4)	7 [1–10]	100	5.4 (3.0)	6 [0–10]
Logistics and convenience									
Minimal waiting time to receive results	101	7.8 (2.3)	8 [0–10]	101	9.0 (1.3)	10 [3–10]	100	7.9 (1.5)	8 [2–10]
Duration of the test from the time the patient checks in to the time the patient leaves the imaging center	101	7.6 (2.6)	8 [0–10]	101	8.4 (1.7)	8 [2–10]	100	7.5 (1.4)	8 [4–10]
Minimal waiting time from the day the appointment is scheduled until the actual test date	101	7.3 (2.8)	8 [0–10]	101	8.8 (1.2)	9 [5–10]	100	7.8 (1.7)	8 [1–10]
Assistance in preparing insurance-related documents	101	7.0 (3.1)	8 [0–10]	101	8.9 (1.8)	10 [1–10]	100	8.2 (1.5)	8 [3–10]
Complexity of paperwork required	101	7.0 (2.5)	7 [0–10]	101	7.5 (2.2)	8 [0–10]	100	7.3 (1.8)	7 [1–10]
Imaging center technologies									
High-quality results, decreasing likelihood of having to retest	101	9.1 (1.5)	10 [3–10]	101	9.5 (1.3)	10 [2–10]	100	8.9 (1.4)	9 [3–10]
Availability of the latest technology	101	8.9 (1.9)	10 [0–10]	101	7.7 (2.1)	8 [1–10]	100	7.9 (1.7)	8 [2–10]
Discussion of risks and benefits of a stress agent^b^	46	8.8 (1.9)	10 [2–10]	101	8.7 (1.9)	9 [2–10]	100	8.1 (1.6)	8 [3–10]
Option to convert to a drug from an exercise test within the same visit^c^	19	7.4 (3.7)	9 [0–10]	101	8.9 (1.7)	10 [2–10]	100	8.3 (1.3)	8 [5–10]
Availability of a specific stress agent^b^	46	7.2 (3.4)	8 [0–10]	101	6.8 (3.2)	8 [0–10]	100	7.4 (2.3)	8 [0–10]
Diverse list of stress agents^b^	46	7.0 (3.1)	8 [0–10]	101	4.1 (2.8)	4 [0–10]	100	6.5 (2.5)	7 [0–10]
Technology for entertainment during waiting periods	101	6.4 (3.1)	7 [0–10]	101	6.1 (2.6)	6 [0–10]	100	6.0 (2.2)	6 [0–10]
Imaging center accessibility									
Convenience of parking at the imaging center	101	7.9 (2.4)	9 [0–10]	101	9.0 (1.3)	9 [4–10]	100	7.8 (1.7)	8 [1–10]
Signage to ease navigation within the center	101	7.9 (2.5)	8 [0–10]	101	8.6 (1.6)	9 [5–10]	100	7.4 (2.0)	8 [0–10]
Imaging center providing detailed directions to the center	101	7.8 (2.6)	9 [0–10]	101	8.6 (1.6)	9 [4–10]	100	7.8 (1.6)	8 [2–10]
Assistance and infrastructure available for patients with impaired mobility	101	7.6 (3.1)	9 [0–10]	101	8.8 (1.7)	9 [2–10]	100	8.0 (1.6)	8 [4–10]
Distance of the imaging center from the patient’s home	101	7.6 (2.4)	8 [0–10]	101	8.0 (1.9)	8 [0–10]	100	7.6 (1.5)	8 [3–10]
Center staff and atmosphere									
Highly skilled and knowledgeable staff	101	9.3 (1.6)	10 [0–10]	101	9.7 (0.7)	10 [7–10]	100	8.7 (1.4)	9 [4–10]
Compassionate and respectful staff	101	9.0 (1.7)	10 [0–10]	101	9.8 (0.5)	10 [7–10]	100	8.9 (1.2)	9 [4–10]
Respect for patient privacy	101	8.8 (1.9)	10 [0–10]	101	9.3 (1.3)	10 [4–10]	100	8.4 (1.5)	9 [4–10]
Comfort of the test	101	8.7 (1.9)	9 [0–10]	101	9.2 (1.0)	9 [5–10]	100	8.3 (1.4)	8 [5–10]
Clean waiting room area*	101	8.7 (1.7)	9 [0–10]	101	9.0 (1.2)	9 [5–10]	100	8.2 (1.4)	8 [4–10]
Quiet waiting room area*	101	7.1 (2.9)	8 [0–10]	101	7.7 (1.6)	8 [4–10]	100	7.3 (1.7)	7 [1–10]
Imaging center providing a snack and drink after the test*	101	5.8 (3.5)	6 [0–10]	101	7.7 (2.0)	8 [1–10]	100	6.0 (2.5)	6 [0–10]
Interior design of the imaging facility	101	5.6 (3.4)	6 [0–10]	101	7.0 (1.9)	7 [2–10]	100	6.4 (2.1)	7 [0–10]

Key: SD, standard deviation; SPECT-MPI, single-photon emission computed tomography myocardial perfusion imaging. 1 physician mentioned accuracy of interpretation, 1 physician mentioned safety facility, and 1 nuclear imaging center staff mentioned physician turnaround time as additional factors that are important to the patient experience with SPECT-MPI testing

^a^Listed factors were among the 27 original factors identified in literature, unless otherwise indicated with an asterisk (“*”) to denote those added as a result of the interviews. The original factor of “clean” and “quiet” waiting room area was separated into 2 factors (based on feedback that these factors are considered independently, rather than as a whole)

^b^All physicians (N = 100), all nuclear imaging center staff (N = 101), and patients who had used a pharmacologic stress agent (N = 46) were asked to rate this factor

^c^All physicians (N = 100), all nuclear imaging center staff (N = 101), and patients who had used a pharmacologic stress agent (N = 19) were asked to rate this factor

### Congruence of Factor Ratings Between Groups

The highest-rated factors were similar across all three groups: high-quality results/decreasing likelihood of having to retest (median 10 for patients and center staff vs 9 for physicians), highly skilled and knowledgeable staff (median 10 for patients and center staff vs 9 for physicians), and compassionate and respectful staff (median 10 for patients and center staff vs 9 for physicians) (Table 2). All three groups also gave similar ratings for five additional factors, including complexity of paperwork required (median 7 for patients and physicians vs 8 for center staff), availability of a specific stress agent (median for all, 8), technology for entertainment during waiting periods (median 7 for patients vs 6 for center staff and physicians), quiet waiting room area (median 8 for patients and center staff vs 7 for physicians), and interior design of the imaging facility (median 6 for patients vs 7 for center staff and physicians).

### Physicians vs Patients

In addition to the aforementioned factors with largely congruent ratings across all three groups, both physicians and patients agreed that providing information regarding how to prepare for the test was important (medians, 9 and 10, respectively; *P *= 0.042) and that the imaging center providing a snack and drink after the test was not as critical to the patient experience (median for both, 6, *P *= 0.702) (Tables 2 and 3). Several differences between groups were noted, however. Physicians significantly underestimated the importance of 12 of the 32 factors to patients, including information regarding what to expect during the test, respect for patient privacy, and availability of the latest technology. Notably, physicians considered independent patient access to results the least important factor to their patients with a median rating of 6, whereas the median rating assigned by patients was 8 (*P *<  0.017). Lastly, there was a high level of rating congruency between PCPs and cardiologists, except for the importance of availability of a specific stress agent, option to convert to exercise test within the same visit, and high-quality test, which PCPs rated significantly lower compared to cardiologists (*P *<  0.017).

**Table 3:** Variance of importance to the patient experience

Factor	Physician vs patient	Imaging center staff vs patient	Physician vs imaging center staff
	*P* value^a^	*P* value^a^	*P* value^a^
Communication and education			
Information regarding what to expect during the test	< 0.017*	0.142	< 0.017*
Explanation of the necessity and objectives of the test	< 0.017*	0.907	< 0.017*
Information regarding how to prepare for the test	0.042	< 0.017*	< 0.017*
Description of what a SPECT-MPI test is	< 0.017*	0.554	< 0.017*
Information regarding what to expect after the test	< 0.017*	0.591	< 0.017*
Information regarding test location and directions to the nuclear imaging center	0.183	< 0.017*	< 0.017*
Independent patient access to results	< 0.017*	0.022	0.030
Logistics and convenience			
Minimal waiting time to receive results	0.265	< 0.017*	< 0.017*
Duration of the test from the time the patient checks in to the time the patient leaves the imaging center	0.023	0.110	< 0.017*
Minimal waiting time from the day the appointment is scheduled until the actual test date	0.953	< 0.017*	< 0.017*
Assistance in preparing insurance-related documents	0.089	< 0.017*	< 0.017*
Complexity of paperwork required	0.654	0.164	0.215
Imaging center technologies			
High-quality results, decreasing likelihood of having to retest	0.098	0.039	< 0.017*
Availability of the latest technology	< 0.017*	< 0.017*	0.930
Discussion of risks and benefits of a stress agent^b^	< 0.017*	0.579	< 0.017*
Option to convert to a drug from an exercise test within the same visit^c^	0.576	0.195	< 0.017*
Availability of a specific stress agent^b^	0.244	0.283	0.896
Diverse list of stress agents^b^	0.072	< 0.017*	< 0.017*
Technology for entertainment during waiting periods	0.034	0.195	0.499
Imaging center accessibility			
Convenience of parking at the imaging center	0.064	< 0.017*	< 0.017*
Signage to ease navigation within the center	< 0.017*	0.088	< 0.017*
Imaging center providing detailed directions to the center	0.087	0.043	< 0.017*
Assistance and infrastructure available for patients with impaired mobility	0.151	0.026	< 0.017*
Distance of the imaging center from the patient’s home	0.079	0.376	< 0.017*
Center staff and atmosphere			
Highly skilled and knowledgeable staff	< 0.017*	0.080	< 0.017*
Compassionate and respectful staff	0.097	< 0.017*	< 0.017*
Respect for patient privacy	< 0.017*	0.053	< 0.017*
Comfort of the test	< 0.017*	0.353	< 0.017*
Clean waiting room area	< 0.017*	0.268	< 0.017*
Quiet waiting room area	0.388	0.904	0.210
Imaging center providing a snack and drink after the test	0.702	< 0.017*	< 0.017*
Interior design of the imaging facility	0.451	0.030	0.057

Key: SD, standard deviation; SPECT-MPI, single-photon emission computed tomography myocardial perfusion imaging. 100 physicians, 101 patients, and 101 nuclear imaging center staff were included in the pairwise comparisons

^a^*P* values were calculated using Wilcoxon rank-sum tests using Bonferroni adjusted alpha levels of 0.017 (0.05/3). *P* values <  0.017 were indicated with an asterisk (“*”)

^b^All physicians (N = 100), all nuclear imaging center staff (N = 101), and patients who had used a pharmacologic stress agent (N = 46) were asked to rate this factor

^c^All physicians (N = 100), all nuclear imaging center staff (N = 101), and patients who had used a pharmacologic stress agent (N = 19) were asked to rate this factor

### Imaging Center Staff vs Patients

Overall, there was more congruence between patients and imaging center staff than between patients and physicians; significant differences were found between patients and imaging center staff on 10 factors, whereas physicians and patients differed significantly on 12 factors (Tables 2 and 3). Imaging center staff and patients both considered information regarding what to expect during the test to be among the top five most important factors to patients (median ratings for both, 10, *P *= 0.142). Their ratings of respect for patient privacy (median for both, 10, *P *= 0.053) and independent patient access to results (median 7 for center staff vs 8 for patients; *P *= 0.022) were likewise consistent; however, the importance of availability of the latest technology and a diverse list of stress agents were significantly more important to patients than imaging center staff had thought (median 10 vs 8 and 8 vs 4, respectively, *P *<  0.017). Although center staff believed that provision of a snack and drink after the test was important to patients, the patients themselves did not consider it as important to their experience (median 8 vs 6; *P *<  0.017).

### Physicians vs Imaging Center Staff

Remarkably little congruence was observed between imaging center staff and physicians, with statistically significant differences in 25 out of 32 ratings (Table 3). Except for a diverse list of stress agents, imaging center staff rated the other 24 differing factors significantly higher compared to physicians. Although the scores differed from each other, both imaging center staff and physicians rated information regarding how to prepare for the test and respect for patient privacy among the top five most important factors related to the patient experience. Independent patient access to results, the least important factor to physicians (median 6), was rated similarly by imaging center staff (median 7).

## Discussion

In this study, which identified and assessed 32 factors that influence patients’ experience during the SPECT-MPI testing process in the US, we found a high level of agreement on the importance of the patient experience between patients and imaging center staff, with information regarding expectations during testing and respect for patient privacy rated highly by both groups. Conversely, physicians viewed these same factors and some others as being of less importance to patients, with overall lower congruence between patients and physicians relative to patients and imaging center staff. In addition to high-quality results with a lower likelihood of having to retest, all participating groups in the study considered compassionate and respectful staff, as well as highly skilled and knowledgeable staff to be among the top most important factors for the patient experience.

The purpose of this study was to gain insight into the patient experience according to the perspective of various stakeholders involved in the SPECT-MPI process, and to assess the congruence of these stakeholder perceptions of patient experience with that of the patients’ opinions. Imaging center staff involved in this study included physicians and other staff who are responsible for the operation and leadership of the laboratory where testing took place. Overall, while direct clinical and diagnostic factors were considered important to the patient experience by patients, imaging center staff, and physicians, all three groups considered non-clinical factors to be highly important to the patient experience as well. The data therefore support previous literature finding patients to be highly concerned with non-clinical factors such as staff behavior and communications.^11^,^12^

While earlier studies have focused on patient and physician perspectives,^11^-^13^ our study also evaluated factors from the perspective of imaging center staff, for whom incongruence with physicians was evident (given their statistical differences on 25 factors). The congruence observed between patients and imaging center staff is consistent with a shared experience between these groups. With respect to SPECT-MPI, imaging center operators have more patient contact and interaction than physicians, which can positively or negatively impact the likelihood that the patient would want to return to that lab. Our findings suggest that imaging center staff may underestimate the extent to which patients are concerned with technical aspects of the procedure, specifically that the imaging center have the latest technology and a diverse list of stress test agents available, while overestimating the importance of facility-related aspects of comfort and convenience, such as the provision of a snack or drink before leaving the imaging center. Overall, there was limited incongruence between the patient and center staff and differences were distributed fairly evenly across factor categories, with the largest number of differences observed on logistics and convenience (where center staff overestimated the importance to patients on 3 of the 5 factors). This was in contrast to the physicians, who not only viewed the availability of the latest technology as being of less importance than it was for patients but also lacked agreement with patients on expectations during testing and patient privacy. This is not necessarily surprising given that physician exposure to the SPECT-MPI testing process is minimal in clinical practice, especially for PCPs, who represented half of the physicians in our sample. While independent patient access to test results was not among the top five important factors to patients, it was ranked at a higher level of importance by patients (median rating, 8) relative to both physicians and imaging center staff (median rating of 6 and 7, respectively). Together with the patient-placed importance on both the latest technology and the diversity of stress agent selection, this finding suggests that patients approach SPECT-MPI testing from a more technical perspective than previously appreciated; whether the education level of the patient sample (> 80% of whom had at least some post-high school education and approximately 50% had at least a bachelor’s degree) contributed to this perspective is unknown, however. Despite advancements with electronic medical records and patient access portals, only half of the patients believed that they had independent access to their results. From the physician perspective, approximately 1 in 3 patients was aware of the results before reviewing them with the ordering physician, suggesting that improvements in patient awareness of their accessibility to the test results may improve the patient experience when undergoing nuclear imaging. Additionally, patients tended to rate “what to expect during the test” higher than referring physicians, suggesting that patient education may be an area to target in improving the patient experience. Enhancements in physicians’ knowledge of the quality and processes of imaging centers to which they refer their patients may also contribute to improvements in patients’ experience. Furthermore, given the similar insights between patients and imaging center staff, imaging center staff should be regarded as essential components of a team-based approach to patient care and vital to SPECT-MPI program/process optimization and to maximizing referrals to the imaging center.

The design of this study allowed for the assessment of the relative importance of factors within each respondent group, as well as the assessment of congruency across groups. This provides a broader insight into what matters to the patient during the SPECT-MPI testing process. Ultimately, this multifaceted approach provides comprehensive information on ways to improve the patient experience with SPECT-MPI testing.

We also acknowledge limitations to this survey study. Although the data were derived from a large population, the use of a panel-based survey may limit generalizability. We found that while patients and center operators represented similar geographic areas, the physicians were much more highly concentrated in suburban areas, which may have contributed to incongruence on some factors. Some additional confounders include differences in educational level and gender ratio, with more women in the imaging center staff and the patient populations relative to the physicians surveyed. The assessment of the patient experience was limited to the included factors identified in the initial phase of this study; it is possible there are additional factors that could influence a patient’s experience. We also acknowledge that some differences between groups were small, possibly reflecting a non-linear treatment of the 11-point scale, such as respondents avoiding extreme points of the scale. Although it is possible that individual respondents may have treated the rating scale differently, we attempted to mitigate these challenges by providing written descriptions for the highest and lowest points on the scale (i.e., “not at all important” to “extremely important”) to help standardize interpretations across respondents. Finally, while the current study included over 300 participants (100 in each of the three groups), evaluating the patient experience among larger populations would allow for exploring differences in factors among various patient subgroups (e.g., by gender, age) and will be considered in future research; additionally, combining the evaluation of factors that impact the patient experience with the measure of other outcomes, such as safety and resource utilization, would provide additional insights into how the patient experience may impact overall patient outcomes.

In conclusion, the findings of this study demonstrate areas for improvement in the patient experience with SPECT-MPI that could lead to improvements in the quality and value of SPECT-MPI overall. Despite congruence in the importance of some factors that contribute to the patient experience (i.e., high-quality results/lower likelihood of having to retest, compassionate and respectful staff, highly skilled and knowledgeable staff) across the three groups, the lack of congruence between patients and referring physicians on the importance of other factors suggests the need for physicians to have a better understanding of the SPECT-MPI process at the centers to which they refer patients.

## New Knowledge Gained

This study was designed to address the paucity of available information for improving the patient experience with SPECT-MPI for diagnosing and assessing CAD, gaining insights from not only patients and physicians but also imaging center staff. The findings show that while high-quality results/decreasing likelihood of having to retest and favorable staff attributes (highly skilled and knowledgeable, compassionate, and respectful) are of highest importance to patients, physicians, and imaging center staff underestimated the extent to which patients also place importance on more technical aspects of the procedure. In the assessment of congruence, imaging center staff were most aligned with patients with respect to identifying factors of patient importance, supporting their integral role in SPECT-MPI program/process optimization.

## Electronic Supplementary Material

Below is the link to the electronic supplementary material.

Supplementary material 1 (DOCX 31 kb)

Supplementary material 2 (PPTX 706 kb)

## Abbreviations

CAD: Coronary artery disease
PCP: Primary care physician
SPECT-MPI: Single-photon emission computed tomography myocardial perfusion imaging
