# Supplementary material for: Factors that impact a patient’s experience when undergoing single-photon emission computed tomography myocardial perfusion imaging (SPECT-MPI) in the US: A survey of patients, imaging center staff, and physicians
Source: J Nucl Cardiol. 2019 Aug 29;28(4):1507–18. doi: 10.1007/s12350-019-01863-0 (PMC8421274; doi:10.1007/s12350-019-01863-0)
Supplement: Supplementary file 1 — Supplementary material 1 (DOCX 31 kb) [file 12350_2019_1863_MOESM1_ESM.docx]

# Appendix

Supplementary Table S1. Description of care process – Patient

|  | **N = 101** | |
| --- | --- | --- |
| **Communication and education** |  | |
| **Information provided to patients prior to the test,^a^ n (%)** |  |  |
| Description of what a SPECT-MPI test is | 74 | (73.3%) |
| Explanation of the necessity and objectives of the test | 72 | (71.3%) |
| Information regarding how to prepare for the test | 81 | (80.2%) |
| Information regarding test location and directions to the nuclear  imaging center | 76 | (75.2%) |
| Information regarding what to expect during the test | 85 | (84.2%) |
| Information regarding what to expect after the test | 68 | (67.3%) |
| None of the above | 2 | (2.0%) |
| Unknown | 1 | (1.0%) |
| **Patients have independent access to results,^b^ n (%)** |  |  |
| Yes | 42 | (41.6%) |
| No | 51 | (50.5%) |
| Unknown | 8 | (7.9%) |
| **Logistics and convenience** |  | |
| **Entities that assist in completing preauthorization forms, n (%)** |  |  |
| Nuclear imaging center | 14 | (13.9%) |
| Ordering physician's office | 20 | (19.8%) |
| Nuclear imaging center and ordering physician's office | 36 | (35.6%) |
| Neither the nuclear imaging center nor the ordering physician's office | 30 | (29.7%) |
| Unknown | 1 | (1.0%) |
| **Duration of the test appointment (hours),^c^ mean (SD) [range]** | 3.4 | (3.1) [0-23] |
| Unknown, n (%) | 18 | (17.8%) |
| **Imaging center technologies** |  | |
| **Patient had a retest ordered due to unclear test results, n (%)** |  |  |
| Yes | 11 | (10.9%) |
| No | 87 | (86.1%) |
| Unknown | 3 | (3.0%) |
| **Patient used a stress agent instead of exercising, n (%)** |  |  |
| Yes | 46 | (45.5%) |
| No | 46 | (45.5%) |
| Unknown | 9 | (8.8%) |
| **Stress agent used by patient^d^ (N = 46), n (%)** |  |  |
| Dobutamine (Dobutrex^®^) | 3 | (6.5%) |
| Adenosine (Adenoscan^®^) | 6 | (13.0%) |
| Regadenoson (Lexiscan^®^) | 8 | (17.4%) |
| Dipyridamole (Persantine^®^) | 3 | (6.5%) |
| Other | 0 | (0.0%) |
| Unknown | 26 | (56.5%) |
| **Patient initially planned to perform exercise and transitioned to a stress agent^d^ (N = 46), n (%)** |  |  |
| Yes | 19 | (41.3%) |
| No | 24 | (52.2%) |
| Unknown | 3 | (6.5%) |
| **Patient had the option to convert to a stress agent from an exercise test within the same visit^e^ (N = 19), n (%)** |  |  |
| Yes | 15 | (78.9%) |
| No | 4 | (21.1%) |
| Unknown | 0 | (0.0%) |
| Key: SPECT-MPI, single-photon emission computed tomography myocardial perfusion imaging; SD, standard deviation. | | |
| ^a^ Respondent could select multiple responses. | | |
| ^b^ Independent access refers to the ability of the patient to see the results before going over the results with their physician—for example, through an online patient portal. | | |
| ^c^ Duration was reported from the time the patient checked in to the time the patient left the imaging center. Summary of duration was based on responses from 83 patients who claimed to know the duration of the test. | | |
| ^d^ Only patients who had used a pharmacologic stress agent were asked to provide this information (N=46). | | |
| ^e^ Only patients who had to convert from an exercise test to a pharmacologic stress agent were asked to provide this information (N=19). | | |

Supplementary Table S2. Description of care process – Nuclear imaging center staff

|  | **N = 101** | |  |
| --- | --- | --- | --- |
| **Communication and education** |  | |  |
| **Information provided to patients prior to the test, n (%)** | |  |  |
| Description of what a SPECT-MPI test is | | 94 | (93.1%) |
| Explanation of the necessity and objectives of the test | | 89 | (88.1%) |
| Information regarding how to prepare for the test | | 101 | (100.0%) |
| Information regarding test location and directions to the nuclear  imaging center | | 99 | (98.0%) |
| Information regarding what to expect during the test | | 92 | (91.1%) |
| Information regarding what to expect after the test | | 77 | (76.2%) |
| None of the above | | 0 | (0.0%) |
| Unknown | | 0 | (0.0%) |
| **Patients have independent access to results,^a^ n (%)** | |  |  |
| Yes | | 51 | (50.5%) |
| No | | 44 | (43.6%) |
| Unknown | | 6 | (5.9%) |
| **Logistics and convenience** | |  | |
| **Entities that assist in completing preauthorization forms, n (%)** | |  |  |
| Nuclear imaging center | | 7 | (6.9%) |
| Ordering physician's office | | 43 | (42.6%) |
| Nuclear imaging center and ordering physician's office | | 47 | (46.5%) |
| Neither the nuclear imaging center nor the ordering physician's office | | 1 | (1.0%) |
| Unknown | | 3 | (3.0%) |
| **Duration of the test appointment (hours),^b^ mean (SD) [range]** | | 3.0 | (0.8) [1-6] |
| Unknown, n (%) | | 5 | (5.0%) |
| **Imaging center technologies** | |  | |
| **Patients are typically aware of which stress agent they are using, n (%)** | |  |  |
| Yes | | 75 | (74.3%) |
| No | | 22 | (21.8%) |
| Unknown | | 4 | (4.0%) |
| **Patients typically have the option to convert to a stress agent from an exercise test within the same visit, n (%)** | |  |  |
| Yes | | 98 | (97.0%) |
| No | | 1 | (1.0%) |
| Unknown | | 2 | (2.0%) |
| **Factors that influence which stress agent(s) the imaging center offers,^c,d^ n (%)** | |  |  |
| Cost of the agent | | 63 | (62.4%) |
| Known side effects of the stress agent | | 88 | (87.1%) |
| Patient preference | | 29 | (28.7%) |
| Other | | 29 | (28.7%) |
| Unknown | | 7 | (6.9%) |
| **Among factors influencing stress agent(s) used above, which one is the most important^e^ (N = 94), n (%)** | |  |  |
| Cost of the agent | | 14 | (14.9%) |
| Known side effects of the stress agent | | 55 | (58.5%) |
| Patient preference | | 1 | (1.1%) |
| Other | | 21 | (22.3%) |
| Unsure | | 3 | (3.2%) |
| **Pharmacologic stress agent(s) used by respondent's imaging center,^c^ n (%)** | |  |  |
| Dobutamine (Dobutrex^®^) | | 53 | (52.5%) |
| Adenosine (Adenoscan^®^) | | 17 | (16.8%) |
| Regadenoson (Lexiscan^®^) | | 95 | (94.1%) |
| Dipyridamole (Persantine^®^) | | 11 | (10.9%) |
| Other | | 1 | (1.0%) |
| Unknown | | 0 | (0.0%) |
| Key: SPECT-MPI, single-photon emission computed tomography myocardial perfusion imaging;  \|SD, standard deviation. | | | |
| ^a^ Independent access refers to the ability of the patient to see the results before going over the results with their physician—for example, through an online patient portal. | | | |
| ^b^ Duration was reported from the time the patient checked in to the time the patient left the imaging center. Summary of duration was based on responses from 96 nuclear imaging center staff who claimed to know the duration of the test.  ^c^ Respondent could select multiple responses. | | | |
| ^d^ Additional factors that influenced the choice of agent included physician preference (N=11), ease of use (N=7), patient health history (N=3), insurance coverage (N=2), availability of agent (N=2), and accuracy of results (N=2). | | | |
| ^e^ Only nuclear imaging center staff who claimed to know which factors influenced stress agent use (N=94) were asked to provide information on the most important factor in determining which stress agent is used. | | | |

Supplementary Table S3. Description of care process – Physician

|  | **N = 100** | |
| --- | --- | --- |
| **Communication and education** |  | |
| **Information provided to patients prior to the test, n (%)** |  |  |
| Description of what a SPECT-MPI test is | 76 | (76.0%) |
| Explanation of the necessity and objectives of the test | 75 | (75.0%) |
| Information regarding how to prepare for the test | 83 | (83.0%) |
| Information regarding test location and directions to the nuclear  imaging center | 82 | (82.0%) |
| Information regarding what to expect during the test | 73 | (73.0%) |
| Information regarding what to expect after the test | 53 | (53.0%) |
| None of the above | 1 | (1.0%) |
| Unknown | 3 | (3.0%) |
| **Patients have independent access to results,^a^ n (%)** |  |  |
| Yes | 27 | (27.0%) |
| No | 68 | (68.0%) |
| Unknown | 5 | (5.0%) |
| **Logistics and convenience** |  | |
| **Entities that assist in completing preauthorization forms, n (%)** |  |  |
| Nuclear imaging center | 13 | (13.0%) |
| Ordering physician's office | 46 | (46.0%) |
| Nuclear imaging center and ordering physician's office | 30 | (30.0%) |
| Neither the nuclear imaging center nor the ordering physician's office | 2 | (2.0%) |
| Unknown | 9 | (9.0%) |
| **Duration of the test appointment (hours),^b^ mean (SD) [range]** | 3.2 | (1.5) [1-10] |
| Unknown, n (%) | 24 | (24.0%) |
| **Imaging center technologies** |  | |
| **Patients are typically aware of which stress agent they are using, n (%)** |  |  |
| Yes | 41 | (41.0%) |
| No | 42 | (42.0%) |
| Unknown | 17 | (17.0%) |
| **Patients typically have the option to convert to a stress agent from an exercise test within the same visit, n (%)** |  |  |
| Yes | 80 | (80.0%) |
| No | 12 | (12.0%) |
| Unknown | 8 | (8.0%) |
| **Factors that influence which stress agent is used,^c,d^ n (%)** |  |  |
| Cost of the agent | 70 | (70.0%) |
| Known side effects of the stress agent | 83 | (83.0%) |
| Patient preference | 37 | (37.0%) |
| Imaging center preference | 85 | (85.0%) |
| Other | 4 | (4.0%) |
| Unknown | 2 | (2.0%) |
| **Among factors influencing stress agent(s) used above, which one is the most important^e^ (N = 98), n (%)** |  |  |
| Cost of the agent | 16 | (16.3%) |
| Known side effects of the stress agent | 33 | (33.7%) |
| Patient preference | 7 | (7.1%) |
| Imaging center preference | 38 | (38.8%) |
| Other | 2 | (2.0%) |
| Unsure | 2 | (2.0%) |
| **Physician-preferred pharmacologic stress agent, n (%)** |  |  |
| Dobutamine (Dobutrex^®^) | 15 | (15.0%) |
| Adenosine (Adenoscan^®^) | 12 | (12.0%) |
| Regadenoson (Lexiscan^®^) | 56 | (56.0%) |
| Dipyridamole (Persantine^®^) | 7 | (7.0%) |
| Imaging center determines which stress agent to use | 9 | (9.0%) |
| Other | 0 | (0.0%) |
| Unknown | 1 | (1.0%) |
| Key: SPECT-MPI, single-photon emission computed tomography myocardial perfusion imaging;  SD, standard deviation. | | |
| ^a^ Independent access refers to the ability of the patient to see the results before going over the results with their physician—for example, through an online patient portal. | | |
| ^b^ Duration was reported from the time the patient checked in to the time the patient left the imaging center. Summary of duration was based on responses from 76 physicians who claimed to know the duration of the test.  ^c^ Respondent could select multiple responses. | | |
| ^d^ Additional factors that influenced the choice of agent included availability of agent (N=2), ease of use (N=1), and patient health history (N=1). | | |
| ^e^ Only physicians who claimed to know which factors influenced stress agent use (N=98) were asked to provide information on the most important factor in determining which stress agent is used. | | |
