# Supplementary material for: Factors that impact a patient’s experience when undergoing single-photon emission computed tomography myocardial perfusion imaging (SPECT-MPI) in the US: A survey of patients, imaging center staff, and physicians
Source: J Nucl Cardiol. 2019 Aug 29;28(4):1507–18. doi: 10.1007/s12350-019-01863-0 (PMC8421274; doi:10.1007/s12350-019-01863-0)
Supplement: Supplementary file 2 — Supplementary material 2 (PPTX 706 kb) [file 12350_2019_1863_MOESM2_ESM.pptx]

## Slide 1
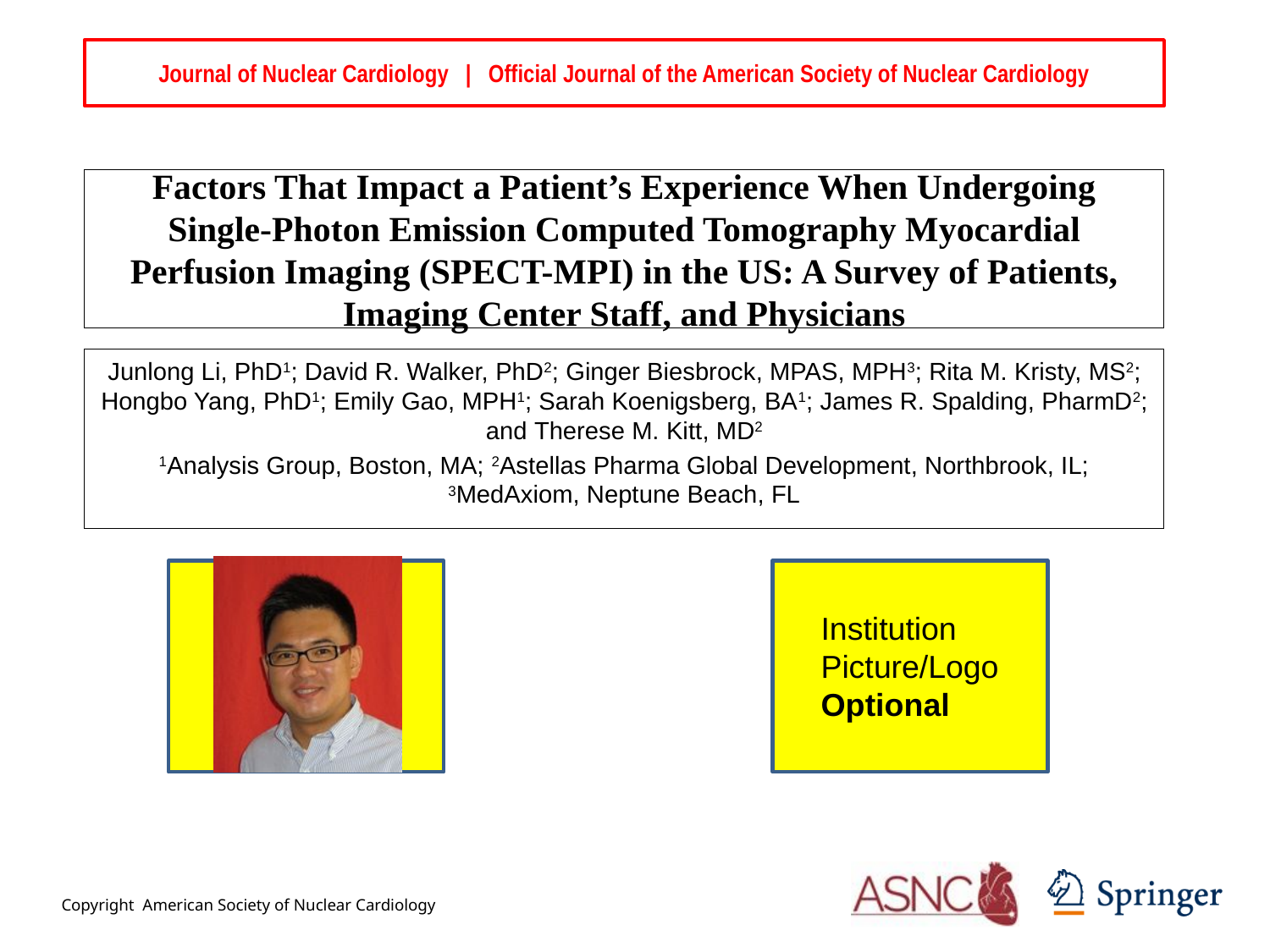

Journal of Nuclear Cardiology | Official Journal of the American Society of Nuclear Cardiology
# Factors That Impact a Patient’s Experience When Undergoing Single-Photon Emission Computed Tomography Myocardial Perfusion Imaging (SPECT-MPI) in the US: A Survey of Patients, Imaging Center Staff, and Physicians
Junlong Li, PhD1; David R. Walker, PhD2; Ginger Biesbrock, MPAS, MPH3; Rita M. Kristy, MS2; Hongbo Yang, PhD1; Emily Gao, MPH1; Sarah Koenigsberg, BA1; James R. Spalding, PharmD2; and Therese M. Kitt, MD2
1Analysis Group, Boston, MA; 2Astellas Pharma Global Development, Northbrook, IL; 3MedAxiom, Neptune Beach, FL
Head shot of author
required
Institution
Picture/Logo
Optional
Copyright American Society of Nuclear Cardiology

## Slide 2
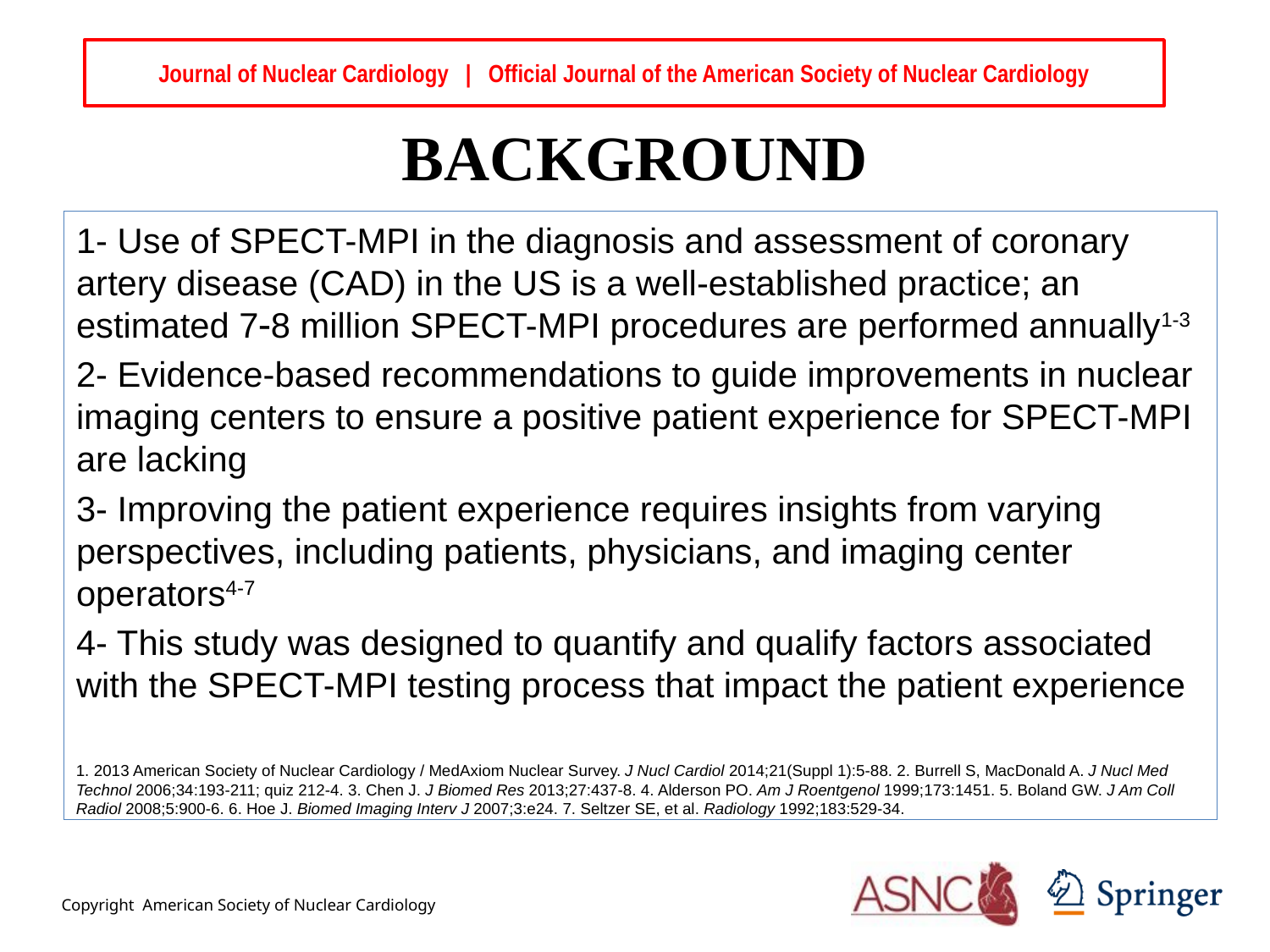

Journal of Nuclear Cardiology | Official Journal of the American Society of Nuclear Cardiology
# BACKGROUND
1- Use of SPECT-MPI in the diagnosis and assessment of coronary artery disease (CAD) in the US is a well-established practice; an estimated 7−8 million SPECT-MPI procedures are performed annually1-3
2- Evidence-based recommendations to guide improvements in nuclear imaging centers to ensure a positive patient experience for SPECT-MPI are lacking
3- Improving the patient experience requires insights from varying perspectives, including patients, physicians, and imaging center operators4-7
4- This study was designed to quantify and qualify factors associated with the SPECT-MPI testing process that impact the patient experience
1. 2013 American Society of Nuclear Cardiology / MedAxiom Nuclear Survey. J Nucl Cardiol 2014;21(Suppl 1):5-88. 2. Burrell S, MacDonald A. J Nucl Med Technol 2006;34:193-211; quiz 212-4. 3. Chen J. J Biomed Res 2013;27:437-8. 4. Alderson PO. Am J Roentgenol 1999;173:1451. 5. Boland GW. J Am Coll Radiol 2008;5:900-6. 6. Hoe J. Biomed Imaging Interv J 2007;3:e24. 7. Seltzer SE, et al. Radiology 1992;183:529-34.
Copyright American Society of Nuclear Cardiology

## Slide 3
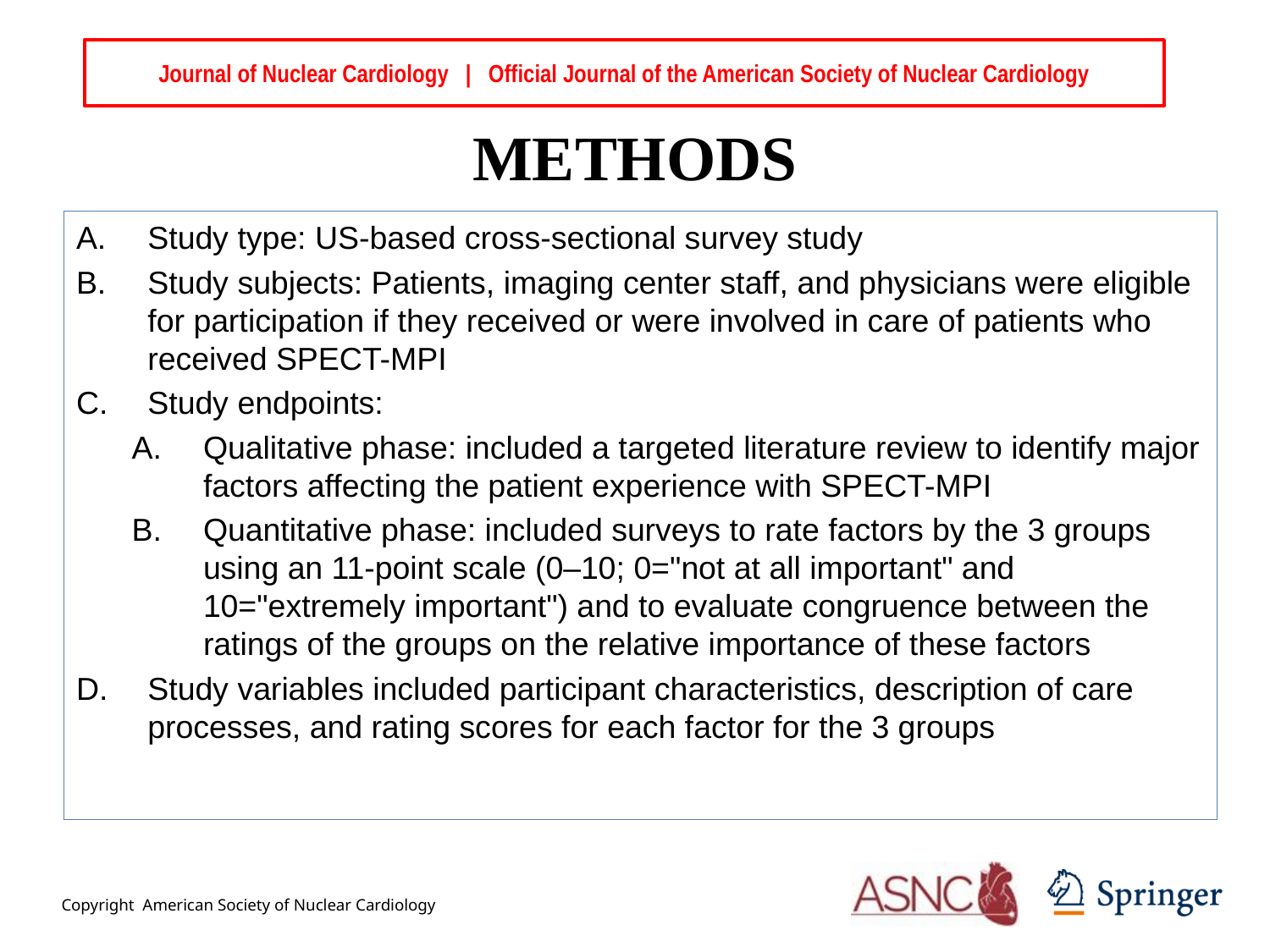

Journal of Nuclear Cardiology | Official Journal of the American Society of Nuclear Cardiology
# METHODS
Study type: US-based cross-sectional survey study
Study subjects: Patients, imaging center staff, and physicians were eligible for participation if they received or were involved in care of patients who received SPECT-MPI
Study endpoints:
Qualitative phase: included a targeted literature review to identify major factors affecting the patient experience with SPECT-MPI
Quantitative phase: included surveys to rate factors by the 3 groups using an 11-point scale (0–10; 0="not at all important" and 10="extremely important") and to evaluate congruence between the ratings of the groups on the relative importance of these factors
Study variables included participant characteristics, description of care processes, and rating scores for each factor for the 3 groups
Copyright American Society of Nuclear Cardiology

## Slide 4
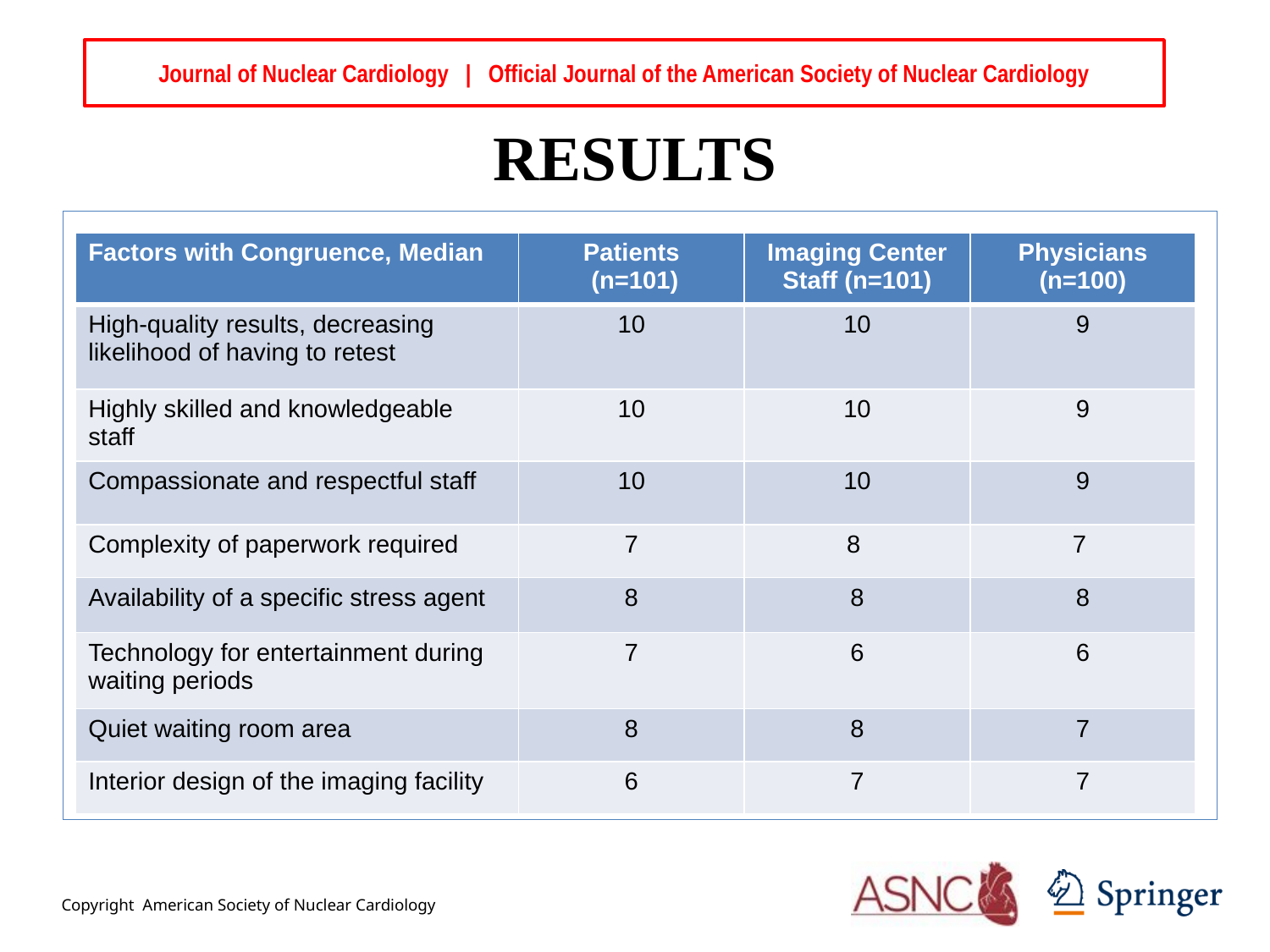

Journal of Nuclear Cardiology | Official Journal of the American Society of Nuclear Cardiology
# RESULTS
| Factors with Congruence, Median | Patients (n=101) | Imaging Center Staff (n=101) | Physicians (n=100) |
| --- | --- | --- | --- |
| High-quality results, decreasing likelihood of having to retest | 10 | 10 | 9 |
| Highly skilled and knowledgeable staff | 10 | 10 | 9 |
| Compassionate and respectful staff | 10 | 10 | 9 |
| Complexity of paperwork required | 7 | 8 | 7 |
| Availability of a specific stress agent | 8 | 8 | 8 |
| Technology for entertainment during waiting periods | 7 | 6 | 6 |
| Quiet waiting room area | 8 | 8 | 7 |
| Interior design of the imaging facility | 6 | 7 | 7 |
Copyright American Society of Nuclear Cardiology

## Slide 5
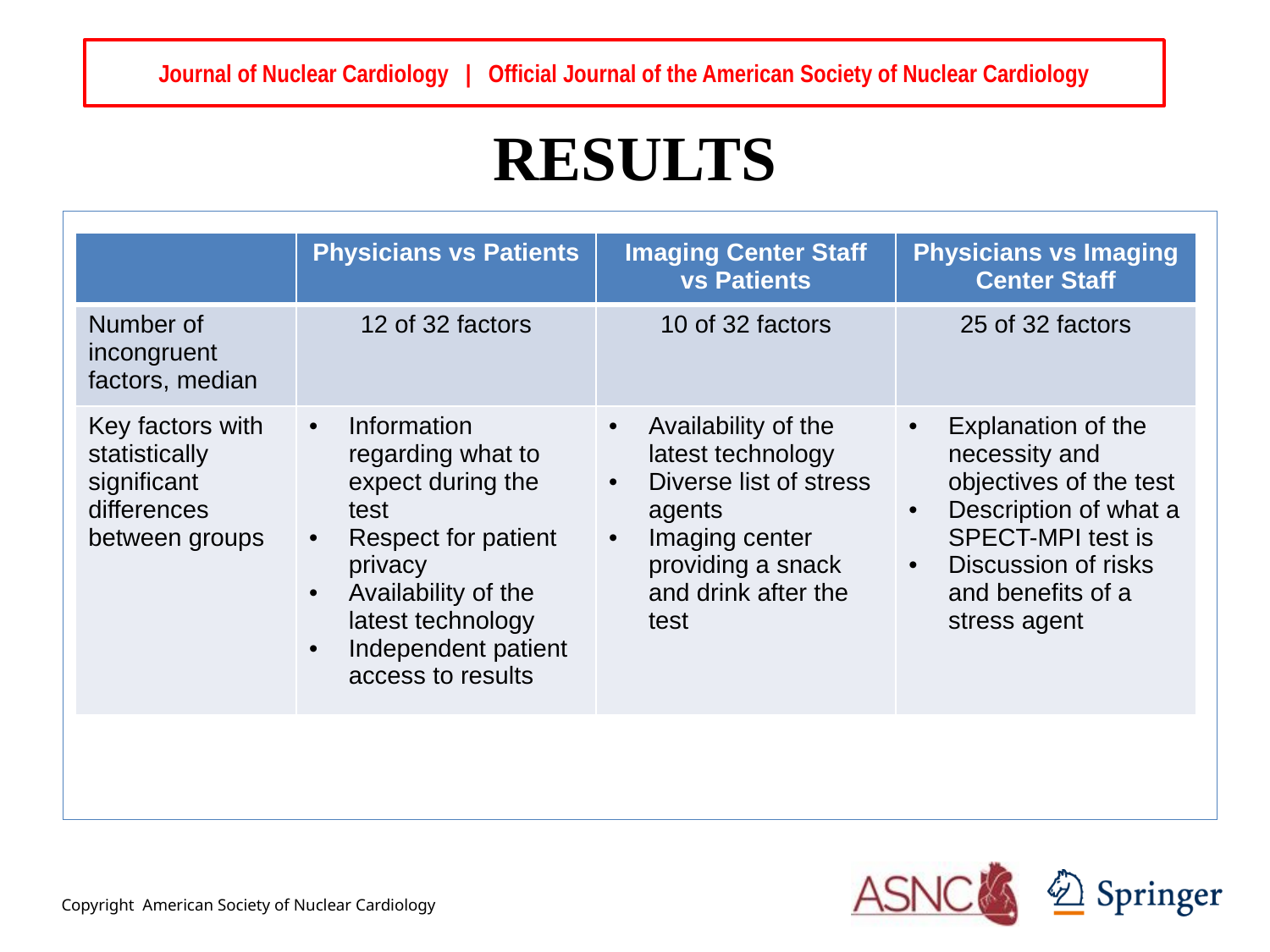

Journal of Nuclear Cardiology | Official Journal of the American Society of Nuclear Cardiology
# RESULTS
| | Physicians vs Patients | Imaging Center Staff vs Patients | Physicians vs Imaging Center Staff |
| --- | --- | --- | --- |
| Number of incongruent factors, median | 12 of 32 factors | 10 of 32 factors | 25 of 32 factors |
| Key factors with statistically significant differences between groups | Information regarding what to expect during the test Respect for patient privacy Availability of the latest technology Independent patient access to results | Availability of the latest technology Diverse list of stress agents Imaging center providing a snack and drink after the test | Explanation of the necessity and objectives of the test Description of what a SPECT-MPI test is Discussion of risks and benefits of a stress agent |
Copyright American Society of Nuclear Cardiology

## Slide 6
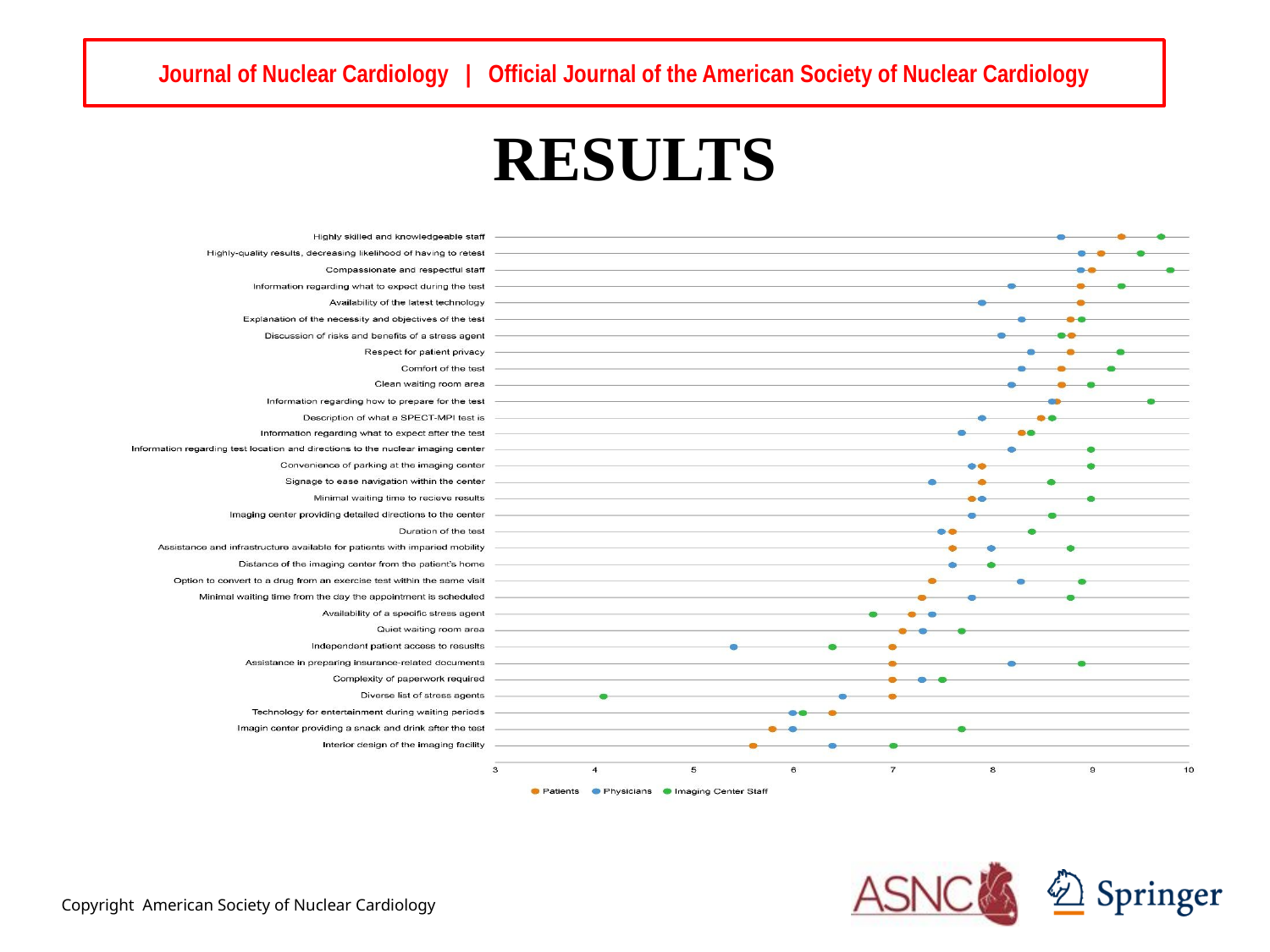

Journal of Nuclear Cardiology | Official Journal of the American Society of Nuclear Cardiology
# RESULTS
Copyright American Society of Nuclear Cardiology

## Slide 7
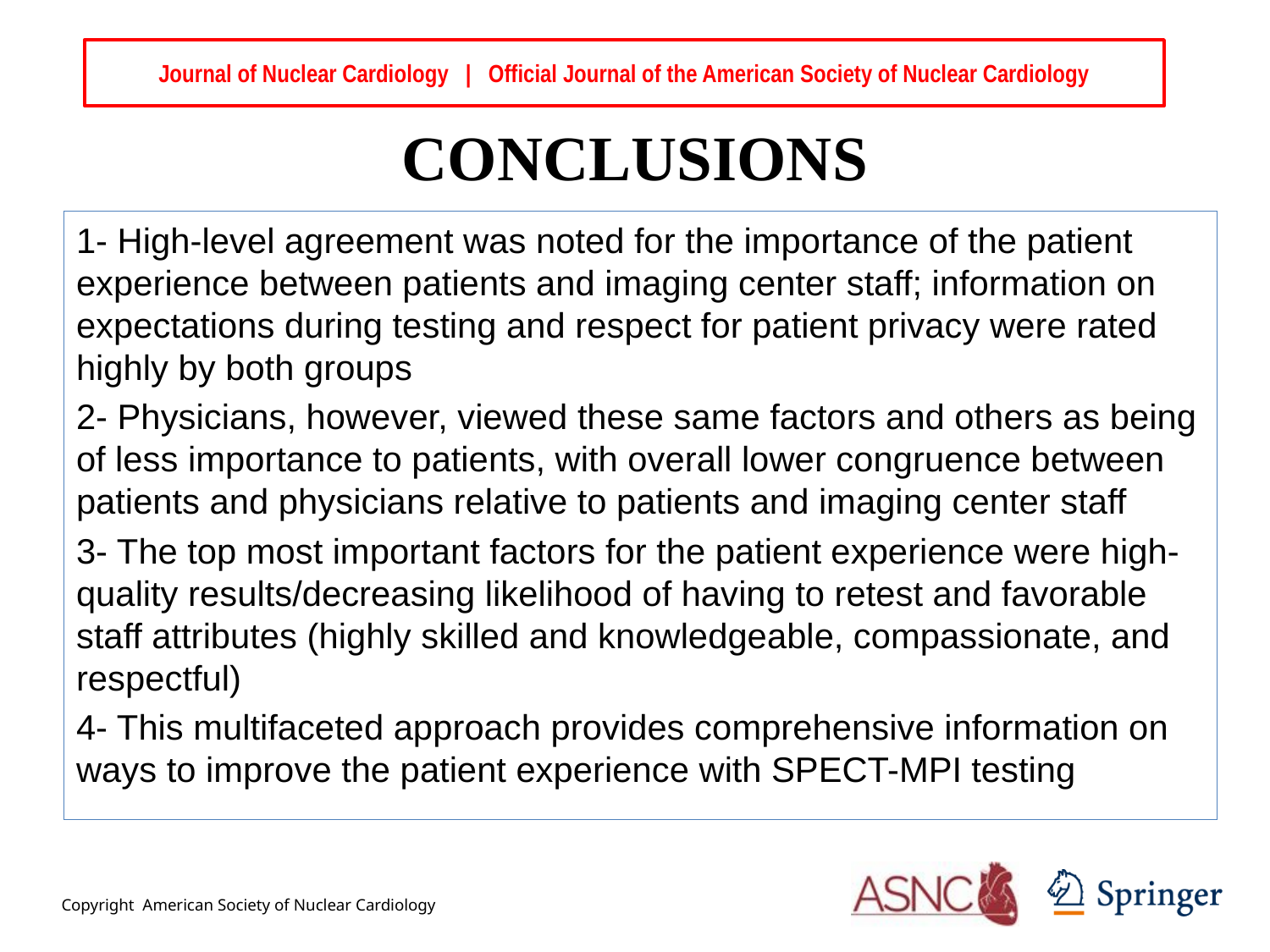

Journal of Nuclear Cardiology | Official Journal of the American Society of Nuclear Cardiology
# CONCLUSIONS
1- High-level agreement was noted for the importance of the patient experience between patients and imaging center staff; information on expectations during testing and respect for patient privacy were rated highly by both groups
2- Physicians, however, viewed these same factors and others as being of less importance to patients, with overall lower congruence between patients and physicians relative to patients and imaging center staff
3- The top most important factors for the patient experience were high-quality results/decreasing likelihood of having to retest and favorable staff attributes (highly skilled and knowledgeable, compassionate, and respectful)
4- This multifaceted approach provides comprehensive information on ways to improve the patient experience with SPECT-MPI testing
Copyright American Society of Nuclear Cardiology
